# Soil N retention and nitrate leaching in three types of dunes in the Mu Us desert of China

**DOI:** 10.1038/srep14222

**Published:** 2015-09-15

**Authors:** Zhao Jin, Yajuan Zhu, Xiangru Li, Yunshe Dong, Zhisheng An

**Affiliations:** 1 State Key Laboratory of Loess and Quaternary Geology, Institute of Earth Environment, Chinese Academy of Sciences, Xi’an 710061, China; 2 Institute of Desertification Studies, Chinese Academy of Forestry, Beijing 100091, China; 3 Institute of Geographical Sciences and Natural Resources Research, Chinese Academy of Sciences, Beijing 100101, China

## Abstract

A large reservoir of soil nitrate in desert subsoil zones has been demonstrated in previous studies; however, information on the subsoil nitrate reservoir and its distribution characteristics in the deserts of China is still limited. This study investigated the distribution patterns of soil total nitrogen (N), nitrate, ammonium, and stable isotopic ratios of ^15^N (δ^15^N) in shallow (1 m) and subsoil (5 m) profiles in three types of dunes in the Mu Us desert of China. We found that soil N retention of the fixed and semi-fixed dunes followed a progressive nutrient depletion pattern in shallow soil profiles, whereas the subsoil nitrate of the fixed, semi-fixed and mobile dunes maintained a conservative accumulation pattern. The results indicate that the subsoil of the Mu Us desert may act as a reservoir of available nitrate. Furthermore, a soil δ^15^N analysis indicate that the nitrate content of the fixed dune is likely derived from soil nitrification, whereas the nitrate content in the mobile dune is derived from atmospheric nitrate deposition. Within the context of looming climate change and intensifying human activities, the subsoil nitrate content in the deserts of northern China could become mobilized and increase environmental risks to groundwater.

Deserts cover one-third of the land surface worldwide and play an important role in the function of global ecosystems and biogeochemical cycling^1^,^2^,^3^,^4^. Desert soils are generally thought to be nutrient poor and low in total nitrogen (N)^1^,^5^. However, recent studies have demonstrated that desert subsoil represents a large reservoir of bioavailable N in the form of nitrate, suggesting that this N pool has been previously overlooked^6^,^7^,^8^,^9^. Investigations of this subsoil N storage could increase estimates of vadose-zone N content by 14 to 71% for warm deserts and arid shrublands worldwide^6^. Moreover, subsoil nitrate may contaminate groundwater and exert further negative effects after land-use or climate change in deserts^6^. Therefore, additional investigations of subsoil nitrate reservoirs are required in the field of desert environmental research.

China contains several of the largest areas of desert and desertified land in the world. The total area of desert in China is estimated at approximately 1.53 × 10^6^ km^2^, and deserts occupy approximately 15.9% of the total national land area^10^,^11^. Numerous studies have suggested that desert subsoil in China could accumulate a large amount of bioavailable N from massive atmospheric nitrate depositions and active N fixation by biological soil crusts^8^,^12^,^13^,^14^. However, to our knowledge, few studies have been conducted to characterize the subsoil nitrate distribution and dynamics in Chinese deserts. Moreover, numerous studies have demonstrated that the climate of northwest China has experienced an increasing warming and wetting trend over the past several decades and suggest that this trend will continue throughout the 21^st^ century^15^,^16^,^17^. Increases in precipitation would lead to additional leaching and mobilization of subsoil nitrate in deserts and heighten the environmental risk to groundwater. Therefore, investigations of the distribution characteristics of subsoil nitrate reservoirs in Chinese deserts are urgently required, and the resulting information will broaden our understanding of desert N cycling.

The Mu Us desert is located in the southeastern region of the Ordos Plateau in northern China and covers an area of 40000 km^2^ 18. Over the past decade, the region has been the most active area of economic growth in China because of its rich coal, oil and natural gas resources. Groundwater is the main water source in the Mu Us desert, and soil nitrate is readily mobilized to the groundwater because of the shallow groundwater table^19^. Moreover, the rapid development of petroleum and coal industries in the region could significantly increase the atmospheric deposition of nitrate and pose an additional potential threat to the groundwater^20^. However, little is known of the distribution patterns of subsoil nitrate in the region, which limits our understanding of whether a subsoil nitrate reservoir occurs in the area and how it might be characterized. In this study, three typical landforms in the Mu Us desert – fixed dune, semi-fixed dune and mobile dune – were selected, and soil samples from shallow profiles (1 m) and subsoil drilling cores (5 m) were analysed to determine the total N, nitrate, ammonium, and δ^15^N (Fig. 1). The objectives of this study were to (1) investigate the characteristics of soil N retention in shallow soil profiles and (2) characterize the distribution patterns of soil nitrate in the subsoil horizon.

## Results

### Plant and soil properties among the three types of dunes

The three types of dunes exhibited considerable differences in plant and soil properties (Table 1). The fixed dune presented the highest vegetation cover, plant species composition, silt and clay content, and bulk density, and *Artemisia ordosica* and *Hedysarum fruticosum* were the dominant species. The semi-fixed dune presented lower vegetation cover, plant species composition, silt and clay content, and bulk density compared with that of the fixed dune, and *A. ordosica* was the dominant species. The mobile dune was barren and presented the highest sand content and lowest soil bulk density.

### Characteristics of soil N retention in the upper 1 m of the soil

**Figure 2:**
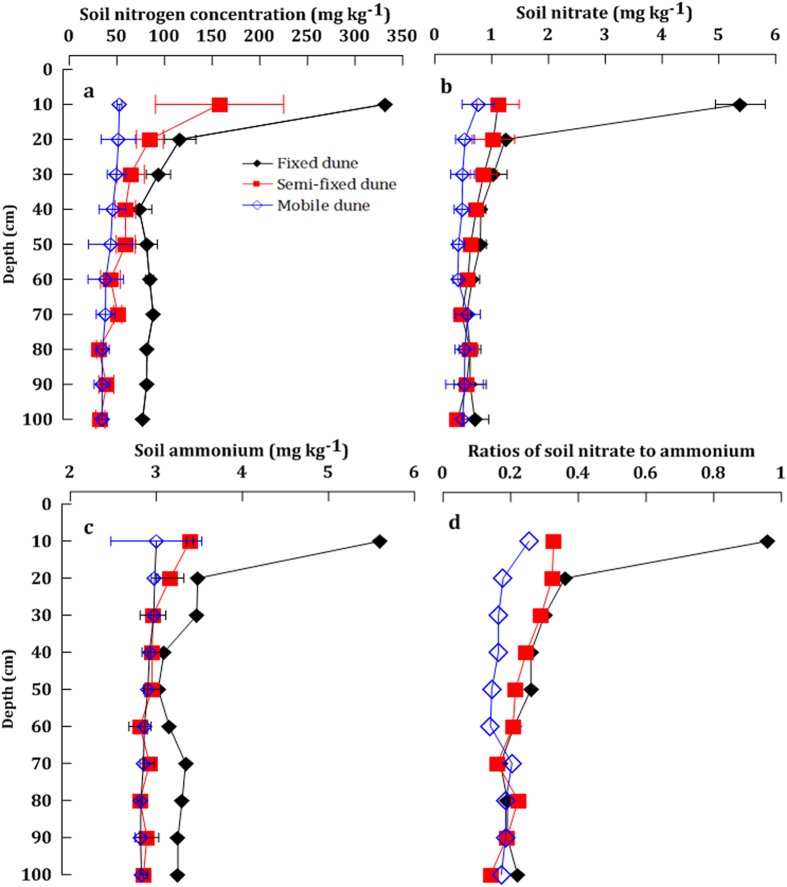
Distribution patterns of total N (a), nitrate (b), ammonium (c) and ratios of nitrate to ammonium (d) in the upper 1 m of the soil.

In the upper 1 m of the soil, the content of soil total N, nitrate and ammonium presented minimal changes with increasing soil depth for the mobile dune, whereas for the fixed and semi-fixed dunes, the soil N content decreased with increasing depth and showed a progressive nutrient depletion pattern (Fig. 2a–c). A statistical analysis demonstrated that the fixed dune has the highest content of soil total N and ammonium among the three types of dunes (total N: *P *= 0.015; ammonium: *P* = 0.012), whereas soil nitrate does not show significant differences (nitrate: *P* = 0.151). Ammonium was the dominant form of soil inorganic N preserved in the upper 1 m of the soil, and the ratios of soil nitrate to ammonium averaged 0.24 for the three types of dunes (Fig. 2d).

### Distribution patterns of soil nitrate in the 5 m soil layer

**Figure 3:**
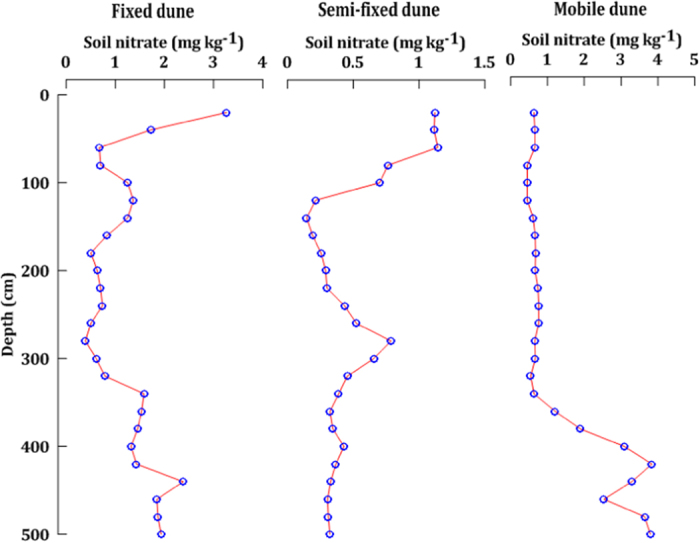
Distribution patterns of soil nitrate in the 5 m soil layer.

In the 5 m soil layer, soil nitrate exhibited obvious pattern of leaching for all of the three types of dunes (Fig. 3). In the mobile dune, the soil nitrate content presented minimal changes within the depth range of 0–3.5 m, whereas below 3.5 m, the soil nitrate content significantly increased and showed an obvious pattern of leaching. In the fixed and semi-fixed dunes, nitrate in the shallow soil profiles showed a progressive nutrient depletion pattern, whereas nitrate in the deep soil horizon exhibited an increasing pattern, although the distribution patterns varied.

### Soil δ^15^N values among the three types of dunes

**Figure 4:**
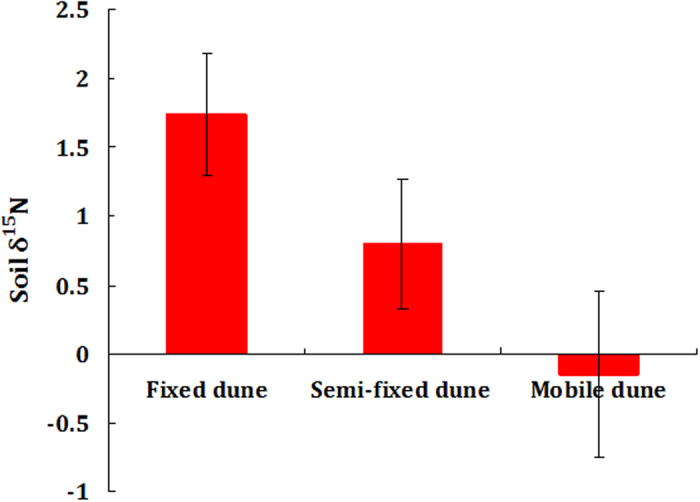
Soil δ^15^N values among the three types of dunes.

The values of δ^15^N in the upper 1 m of the soil exhibited considerable variation among the three types of dunes, averaging 1.74%, 0.80% and −0.15% for the fixed, semi-fixed and mobile dunes, respectively (Fig. 4). A statistical analysis demonstrated that the fixed dune had the highest values of δ^15^N, whereas the mobile dune had the lowest (*P* < 0.001).

## Discussion

The three types of Chinese desert dunes evaluated in this study were found to have large differences in their levels of vegetative cover, which is the primary causal factor for the corresponding differences in the soil N retention and nitrate leaching. In the fixed dune, the vegetation cover was approximately 85%, with a large number of biological soil crusts observed to cover the soil surface^21^,^22^. Dense vegetation cover constitutes a powerful system for biological cycling of soil N and usually leads to progressive nutrient depletion patterns in shallow soil profiles^23^,^24^. Moreover, the dominant species of the fixed dune were *A. ordosica* and *H. fruticosum*. *H. fruticosum* is a species of the legume family, and biological N fixation by *H. fruticosum* and soil crusts also promotes soil N increases at the soil surface^25^,^26^. Moreover, a high degree of vegetation cover and biological soil crust formation can interfere with the atmospheric deposition of N^27^,^28^. These factors contributed to the current situation in which the fixed dunes showed the highest soil total N content and the most obvious pattern of soil N decreases with depth among the three types of dunes (Fig. 2a–c). In contrast, the soil total N, nitrate and ammonium content showed little changes with increasing soil depth for the mobile dune.

Previous studies have demonstrated that the high soil-surface temperatures of deserts (greater than 50 °C), which are driven by solar radiation, can cause abiotic losses of N in the form of NO_*y*_ (all forms of oxidized gaseous N) and NH_3_^29^. Moreover, the soil pH tends to be high in extremely dry areas, which is also a key driver of ammonium loss associated with volatilization^30^. Therefore, ammonium is generally considered difficult to preserve in the desert soil. In this study, we found that ammonium was the dominant form of inorganic N preserved in the upper 1 m of the soil and that the level of soil ammonium was nearly three times higher than that of soil nitrate (Fig. 2d). We conclude that the relatively low temperature and soil pH played a critical role in regulating the soil ammonium retention at the study site. Hu *et al.* (2008) reported that soil ammonium retention is both negatively and significantly correlated with the air temperature and soil pH in the drylands of central East Asia, where low temperature and soil pH are found to correspond to high soil ammonium content^31^. In this study, the annual mean temperature of the area was 6.7 °C and the monthly mean temperatures from April to October range from 7.4–21.9 °C^32^. Moreover, the soil pH of the study area is not too high due to a relatively high rate of precipitation (with an annual average of 345 mm) (Table 1). Numerous studies have reported that low temperature and soil pH could inhibit the ammonium oxidation rate and suppress ammonia volatilization^30^,^33^,^34^,^35^, which are both beneficial to the soil ammonium storage^31^. Moreover, the fixed dune had the highest content of soil ammonium measured among the three types of dunes (*P* = 0.012), indicating that the net soil N accumulation dominates the ecosystem N cycling process as the biological N fixation of the legume species and soil crusts.

In deserts, the storage of available soil N is low^36^,^37^,^38^; as such, the resulting nitrate leaching from desert soils is also expected to be low. We found, however, that the soil nitrate content increased significantly in the subsoil horizon, which indicates that high levels of available soil N in the presence of nitrate have leached into the deeper soil. This result is similar to the findings obtained by Walvoord *et al.* (2003)^6^. In 2003, Walvoord and colleagues discovered the first large reservoir of nitrate beneath desert soils in a western region of the United States. However, Jackson *et al.* (2004) questioned the generality of their results and found no significant increase in the soil nitrate measured in 16 desert soil profiles at depths of 10 m in the Jornada and Sevilleta desert regions of the United States^39^. Recently, Graham *et al.* (2008) reported that large near-surface nitrate pools were found in the soils capped by desert pavement in the Mojave Desert^7^. Therefore, we speculate that subsoil nitrate reservoirs may exist in deserts. In this study, the increase seen in the nitrate content of subsoil indicates the likely presence of a nitrate reservoir in the subsoil zones of the Mu Us desert. However, because of the large differences in the vegetation cover, the three types of dunes showed different patterns of subsoil nitrate distribution. The mobile dune is barren and does not provide physical buffering and biological regulation of vegetation. Consequently, the mobile dune showed a very clear process of nitrate leaching (Fig. 3). In the fixed and semi-fixed dunes, the presence of vegetation was seen to affect the soil N cycling (Figs 2 and 3). As a result, the fixed and semi-fixed dunes had a fluctuating pattern of nitrate leaching (Fig. 3).

Walvoord *et al.* (2003) explained that available soil nitrate is not completely consumed by plants or returned to the atmosphere^6^, which is a possible cause of nitrate leaching in deserts. Moreover, Gebauer and Ehleringer (2000) discovered that desert plants do not necessarily consume water and nutrients simultaneously^40^, which could also contribute to nitrate loss. In this study, the relatively high precipitation rate was found to also promote nitrate leaching, while the soil δ^15^N analysis showed that different dunes may have different sources of nitrate. The fixed dune had the highest values of δ^15^N (1.74%), while the mobile dune had the lowest values of δ^15^N (−0.15%). Nitrate is known to have a more negative δ^15^N value than ammonium^41^,^42^,^43^, indicating a source with increased nitrate content, whereas a more positive value of δ^15^N indicates a source with increased ammonium content^43^. In this study, the highest value of soil δ^15^N was observed in the fixed dune, thus indicating that the biological N fixation of legume species and soil crusts dominates the surface soil N process, producing ammonium and maintaining the nutrient demands of the desert ecosystem. Therefore, nitrate in the fixed dune was most likely to have been derived from soil nitrification. Correspondingly, the lowest soil δ^15^N content was found in the mobile dune, indicating that the soil N in this dune was primarily derived from the atmospheric nitrate deposition.

A number of studies have indicated that changes in climate or land use could mobilize the subsoil nitrate reservoirs that have accumulated over thousands of years in deserts^6^,^7^. The activated subsoil nitrate can contaminate groundwater and adversely affect public water supplies^44^. In the Mu Us desert, the risk of nitrate pollution to the groundwater is high because of the large-scale coal mining operations in the area. Moreover, Hong *et al.* (2014) demonstrated that the rates of summer precipitation in arid eastern Central Asia (to include northwestern China) have increased steadily over the past 8,500 years^45^ and suggested that the trend of a wetter climate would continue in the future^15^,^16^,^45^. Increasing precipitation in the arid regions of China has the potential to mobilize the subsoil nitrate in deserts and increase the environmental risks to groundwater. Therefore, the adverse effects of subsoil nitrate on the groundwater quality should be considered when managing water resources in the arid and semiarid regions of China.

## Methods

### Study area

This study was conducted at the Chinese Academy of Sciences’ Ordos Sandy Grassland Research Station, which is located in the Mu Us desert of Inner Mongolia, China (see Supplementary Fig. S1 online). The area has a typically semiarid climate with marked seasonal and diurnal temperature variations and low precipitation. The annual mean temperature is 6.7 °C, with monthly mean temperatures falling below 5 °C from November to March and ranging from 7.4 °C to 21.9 °C from April to October. The annual mean precipitation is 345 mm, with an annual mean evaporation of 2535 mm. From April to October, the mean precipitation is 322 mm, which accounts for approximately 93% of the annual precipitation^32^. The topography of the area is characterized by sand dunes and desert shrub vegetation, and only a small area of grasslands is distributed in the lowland and upland areas^18^.

In the vicinity of the Ordos Sandy Grassland Research Station, three typical landforms – fixed, semi-fixed, and mobile dunes – were selected as sampling sites. The fixed dune sampling site lies at 111° 11′ 39″ E and 39° 29′ 43″N and is 1313 m above sea level. Vegetation cover accounts for approximately 85% of the area, and the main plant species are *A. ordosica*, *H. fruticosum*, *Hedysarum scoparium* and *Stipa bungeana*^21^,^46^,^47^, with *A. ordosica* and *H. fruticosum* identified as the dominant species (see Supplementary photographs of common plant species in the Ordos Sandy Grassland Research Station online, Figs S2–S66, Zhu Yajuan took and assembled in 2007). The percentages of sand (2–0.02 mm), silt (0.02–0.002 mm) and clay (<0.002 mm) measured at depths of 0–20 cm in the soil profile are 67.85%, 27.97% and 4.10%, respectively^48^. The semi-fixed dune sampling site lies at 111° 8′8″E and 39° 28′51″N and is 1289 m above sea level. Vegetation cover accounts for approximately 25% of the area, and the main plant species are *A. ordosica*, *P. villosa* and *C. komarovii*^21^,^46^,^47^, with *A. ordosica* as the dominant species. The percentages of sand, silt and clay measured at depths of 0−20 cm in the soil profile are 80.54%, 16.94% and 2.52%, respectively^48^. The mobile dune sampling site lies at 111° 11′ 50″ E and 39° 28′ 39″N and is 1317 m above sea level. The mobile dune is barren, and the percentages of sand, silt and clay measured at depths of 0–20 cm in the soil profile are 94.50%, 4.53% and 0.97%, respectively^48^.

### Soil sampling and laboratory analysis

Soil sampling was performed in July, 2013. In the study area, three soil profiles with depths of 0–100 cm were established in each of the fixed, semi-fixed, and mobile dune sampling sites. The distance among the three soil profiles of the fixed dune was approximately 150 m. At each fixed-dune profile, we used a soil corer with a diameter of 5 cm to collect the soil samples. After removing the biological soil crust and litterfall, soil samples were collected at 10-cm intervals to a depth of 100 cm below the soil surface. Soil samples were stored using polyethelene bags and brought to laboratory for further analysis. In the sampling sites of the semi-fixed and mobile dunes, the distance between the soil profiles was observed to be approximately 200 m. Soils were sampled by the same method as used for the fixed dune. In total, 30 soil samples were collected from each sampling site. Moreover, three replicate samples were collected from each profile at intervals of 10 cm to conduct a soil bulk density analysis using a 100 cm^3^ stainless steel cylinder. These shallow soil samples were analysed for soil total N, nitrate, ammonium, δ^15^N and pH. To obtain the subsoil samples, a hand-held auger 6 cm in diameter and 500 cm in length was used to collect soil cores from a depth of 0–500 cm. Soil samples were collected at 20-cm intervals, and 25 soil samples were obtained at each drilling core. Each sampling site contained one core, and 25 soil samples were collected from each sampling site. These subsoil samples were then analysed to measure soil nitrate content.

All of the collected soil samples were air-dried in the laboratory, and any remaining roots were carefully removed. The soil pH was measured in a deionized water suspension (soil:water, 1:2.5) using a DMP-2 mV/pH detector (Quark Ltd., Nanjing, China). For the soil N analysis, the air-dried soil samples were ground in an agate mortar and passed through a 0.15-mm sieve. The soil total N content was measured through micro-Kjeldahl digestion, which was followed by distillation and titration^49^. Soil nitrate and ammonium were extracted with a 2 M KCl solution (soil:solution, 1:5) and then filtered through a 0.45-μm filter^50^. The extracted solutions were analysed to determine their nitrate and ammonium concentrations using a Continuous Flow Analyser. A soil stable N isotopic analysis was performed in the Environmental Stable Isotope Laboratory of the Chinese Academy of Agricultural Sciences. These measurements were performed using an EA-IsoPrime100 Stable Isotope Ratio Mass Spectrometer (Isoprime Ltd, U.K). The ^15^N/^14^N ratio was expressed in δ notation as parts per thousand deviations (%) from the Pee Dee Belemnite (PDB) standard:


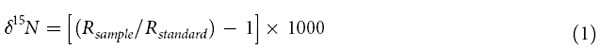


where *R* represents the isotope ratio (^15^N/^14^N) and R_*standard*_ is the ^15^N/^14^N ratio for atmospheric N_2_. The analytical precision for δ^15^N was 0.2%.

### Data analysis

Statistically significant differences in the soil total N, nitrate, ammonium and δ^15^N among the three sampling sites were identified using a one-factor analysis of variance (ANOVA) and least significant difference calculations at an alpha level of 0.05 (*a* < 0.05). All statistical analyses were performed with the Statistical Program for Social Sciences (SPSS 11.0; SPSS Inc., Chicago, IL, USA).

## Additional Information

**How to cite this article**: Jin, Z. *et al.* Soil N retention and nitrate leaching in three types of dunes in the Mu Us desert of China. *Sci. Rep.* **5**, 14222; doi: 10.1038/srep14222 (2015).

## Supplementary Material

Supplementary Information

## Figures and Tables

**Figure 1:**
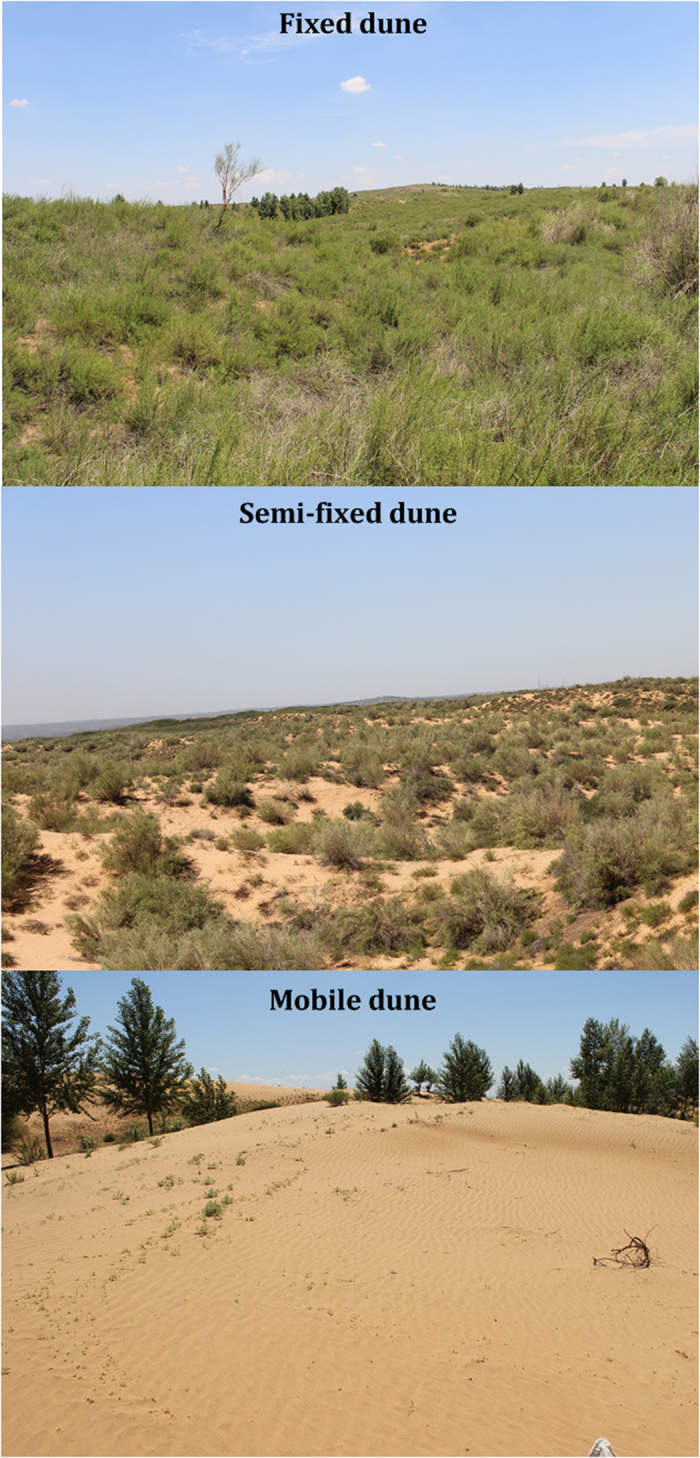
Photographs of the three types of dunes (Zhao Jin took in 2013).

**Table 1:** Plant and soil properties in the fixed, semi-fixed and mobile dunes.

**Plant properties**^21^,^46^,^47^				**Soil properties**^48^				
	**Vegetation cover(%)**	**Plant species composition**	**Dominant species**	**Soil texture (0–20 cm, %, mean ± s.d.)**			**Bulk density (0–100 cm, g cm**^**−3**^**, mean ± s.d.)**	**Soil pH (0−100 cm, mean ± s.d.)**
				**Sand**	**Silt**	**Clay**		
Fixed dune	85	*Artemisia ordosica*, *Hedysarum fruticosum*, *Hedysarum scoparium*, *Stipa bungeana*, *Cleistogenes squarrosa*, *Oxytropis psammochans*, *Lespedeza davurica, Polygala tehuifolia*	*Artemisia ordosica*, *Hedysarum fruticosum*	67.85 ± 10.10^a*^	27.97 ± 9.24^a^	4.10± 1.02^a^	1.66 ± 0.02^a^	7.62 ± 0.17^a^
Semi-fixed dune	25	*Artemisia ordosica*, *Psammochloa villosa,Cynanchum komarovii Chenopodium aristatum,*	*Artemisia ordosica*	80.54 ± 3.49^b^	16.94 ± 3.28^b^	2.52 ± 0.42^c^	1.60 ± 0.05^b^	7. 67 ± 0.22^a^
Mobile dune	0	*none*	*none*	94.50 ± 1.56^c^	4.53 ± 1.11^c^	0.97 ± 0.47^c^	1.53 ± 0.02^c^	7.63 ± 0.09^a^
				*P* < 0.001	*P *< 0.001	*P* < 0.001	*P* < 0.001	*P* = 0.873

Notes: ^*^with different letters within a variable indicates a significant difference.
