# Supplementary material for: Soil N retention and nitrate leaching in three types of dunes in the Mu Us desert of China
Source: Sci Rep. 2015 Sep 15;5:14222. doi: 10.1038/srep14222 (PMC4642556; doi:10.1038/srep14222)
Supplement: Supplementary Information [file srep14222-s1.pdf]

Manuscript title: Soil N retention and nitrate leaching in three types of dunes in the Mu Us desert of China

Author list: Zhao Jin, Yajuan Zhu, Xiangru Li, Yunshe Dong, Zhisheng An

Supplementary figures

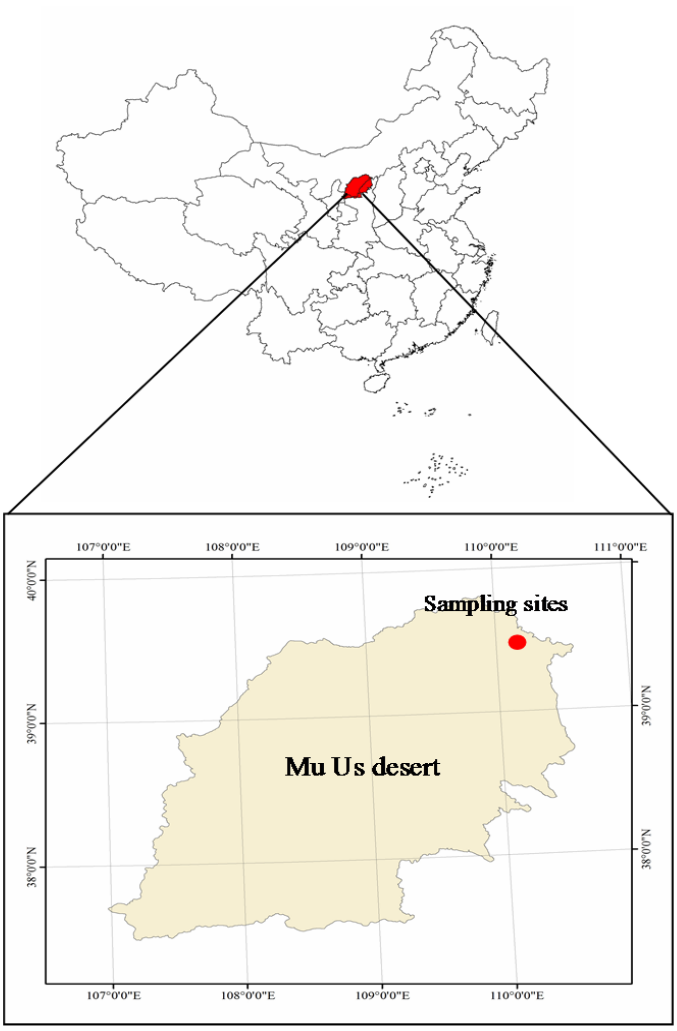

Fig. S1 Map showing the location of the study area in the Mu Us desert of China

(Created by ArcMap version 9.3)

Photographs of common plant species in the Ordos Sandy Grassland Research Station, Chinese Academy of Sciences (Zhu Yajuan took and assembled in 2007)

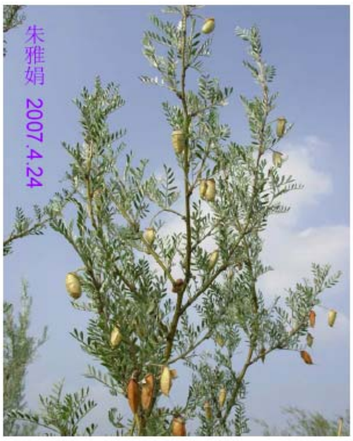

柠条锦鸡儿的果枝

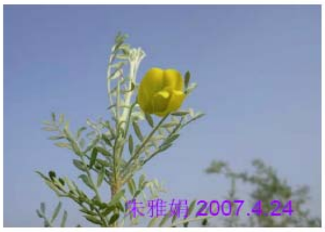

柠条锦鸡儿的花

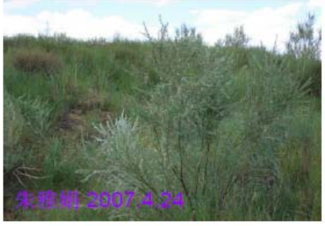

柠条锦鸡儿的全株

Fig. S2 Photographs of *Caragana Korshinskii Kom.*(Zhu Yajuan, 2007)

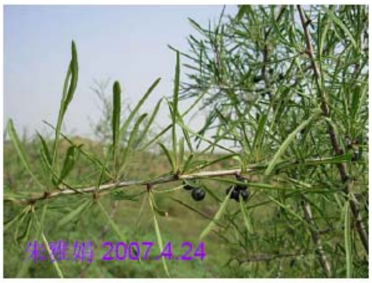

柳叶鼠李的果枝

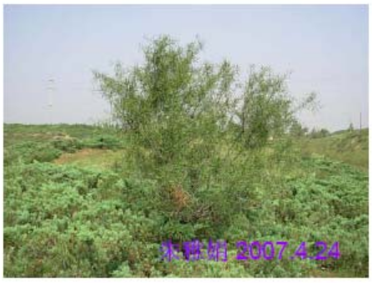

柳叶鼠李的全株

Fig. S3 Photographs of *Rhamnus erythroxylon* Pall. (Zhu Yajuan, 2007)

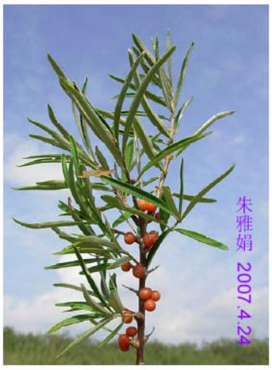

中国沙棘的果枝

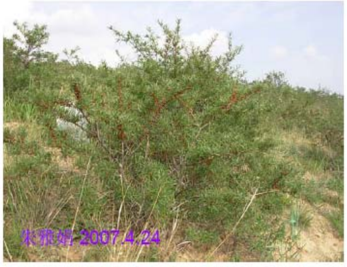

中国沙棘的全株

Fig. S4 Photographs of *Hippophae rhamnoides* L. (Zhu Yajuan, 2007)

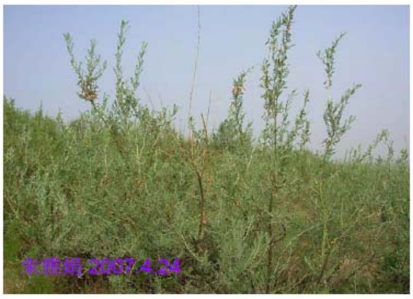

中间锦鸡儿的全株

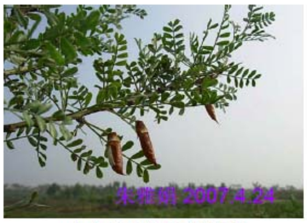

中间锦鸡儿的果枝

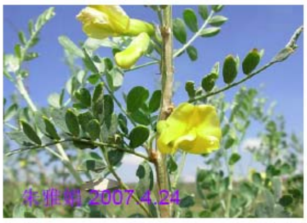

中间锦鸡儿的花枝

Fig. S5 Photographs of *Caragana intermedia* Kuang et al H. C. Fu (Zhu Yajuan, 2007)

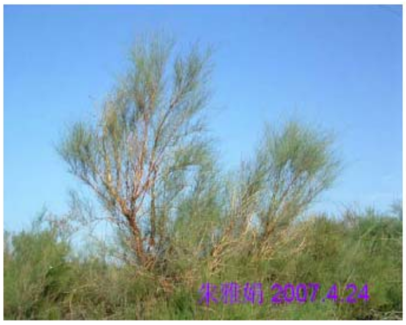

细枝岩黄芪的全株

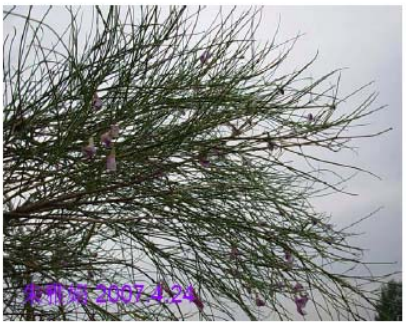

细枝岩黄芪的花枝

Fig. S6 Photographs of *Hedysarum scoparium* Fisch. et al Mey. (Zhu Yajuan, 2007)

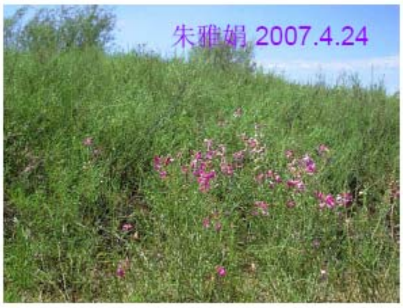

塔洛岩黄芪的全株

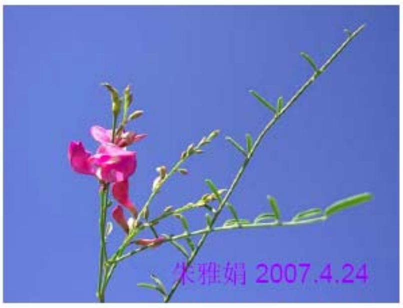

塔洛岩黄芪的花枝

Fig. S7 Photographs of *Hedysarum fruticosum* Maxim. (Zhu Yajuan, 2007)

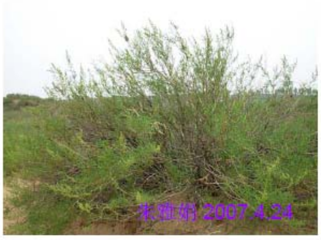

黑沙蒿的全株

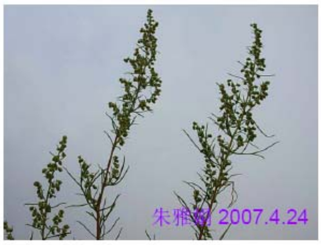

黑沙蒿的花枝

Fig. S8 Photographs of *Artemisia ordosica* Krasch. (Zhu Yajuan, 2007)

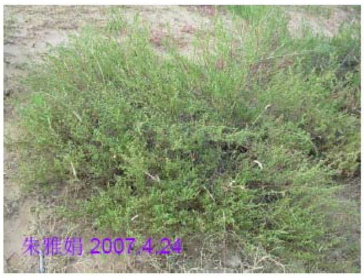

白沙蒿的全株

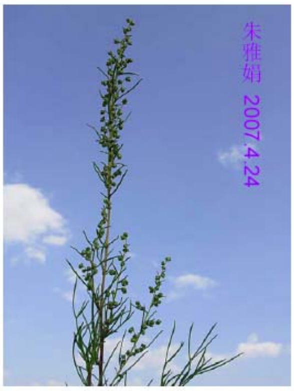

白沙蒿的果枝

Fig. S9 Photographs of *Artemisia sphaerocephala* Krasch. (Zhu Yajuan, 2007)

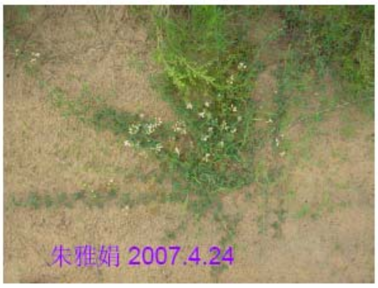

达乌里胡枝子的全株

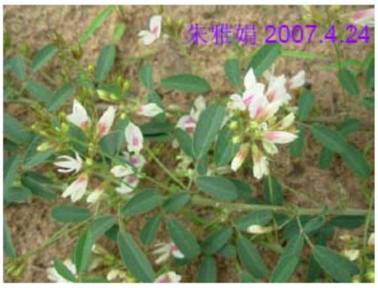

达乌里胡枝子的花

Fig. S10 Photographs of *Lespedeza daurica* (Laxm.) Schindl. (Zhu Yajuan, 2007)

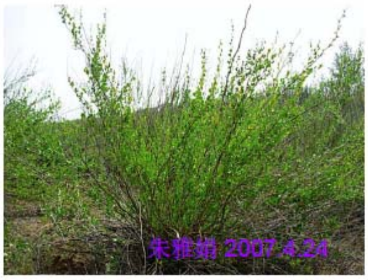

沙木蓼的全株

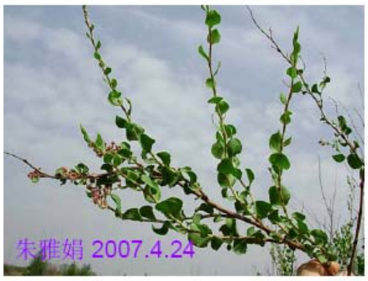

沙木蓼的花枝

Fig. S11 Photographs of *Atraphaxis practeata* A. Los. (Zhu Yajuan, 2007)

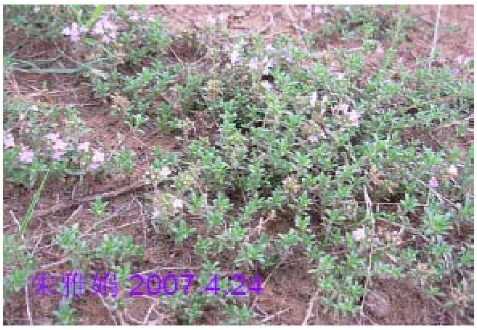

亚洲百里香的全株

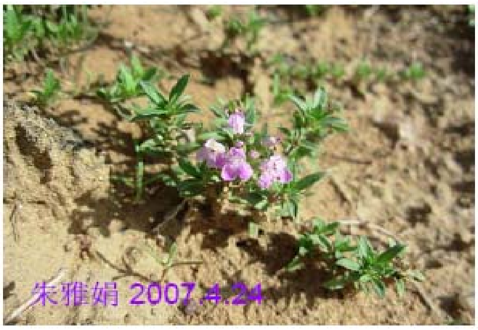

亚洲百里香的花枝

Fig. S12 Photographs of *Thymus mongolicus* Roon. (Zhu Yajuan, 2007)

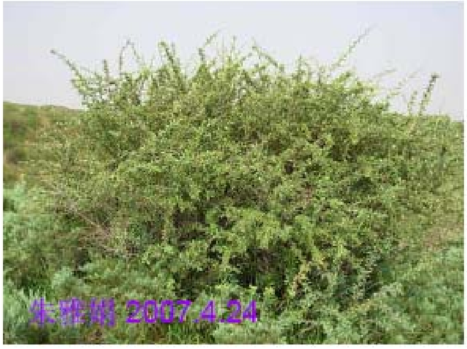

匙叶小檗的全株

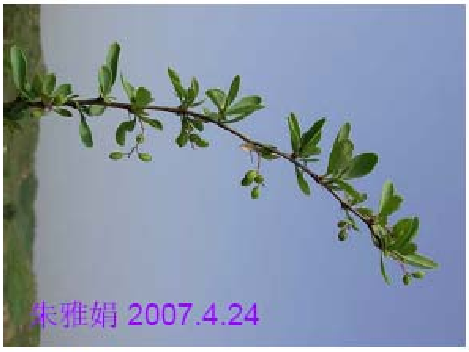

匙叶小檗的果枝

Fig. S13 Photographs of *Berberis vernae* Schneid. (Zhu Yajuan, 2007)

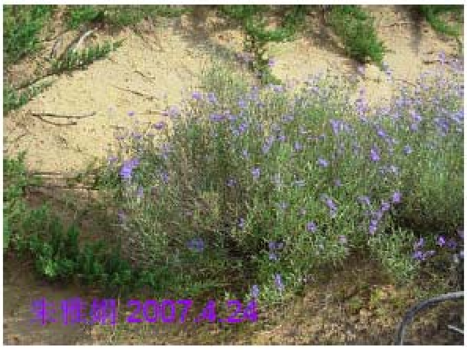

蒙古莸的全株

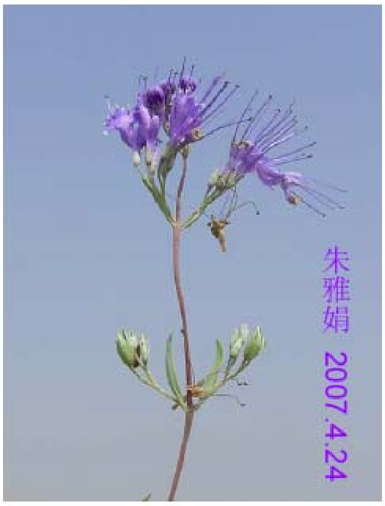

蒙古莸的花枝

Fig. S14 Photographs of *Caryopteris mongholica* Bunge. (Zhu Yajuan, 2007)

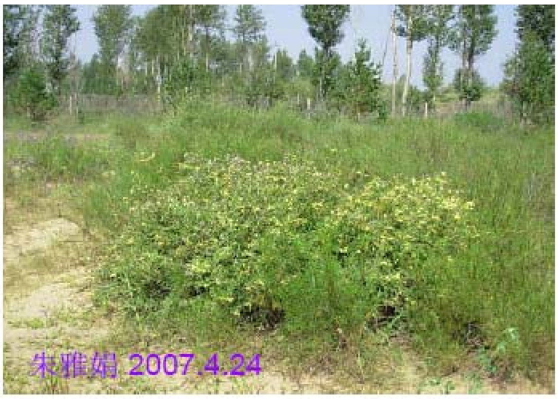

灌木铁线莲的全株

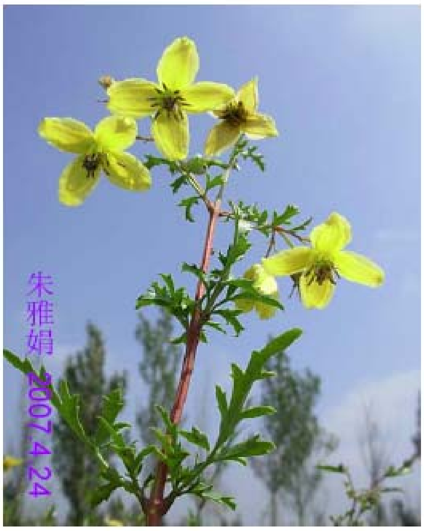

灌木铁线莲的花枝

Fig. S15 Photographs of *Clematis fruticosa* Turcz. (Zhu Yajuan, 2007)

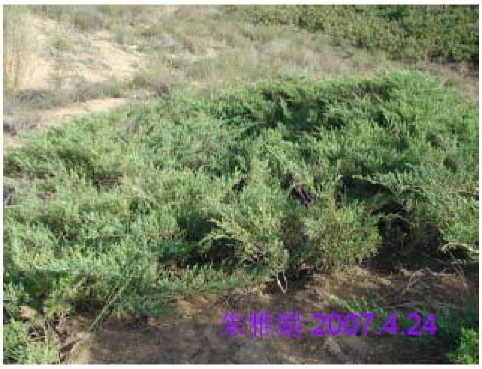

沙地柏的全株

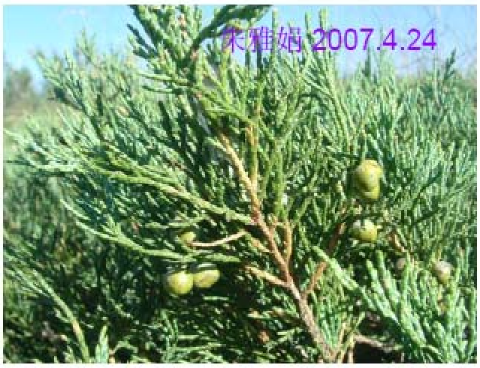

沙地柏的果枝

Fig. S16 Photographs of *Sabina vulgaris* Ant. (Zhu Yajuan, 2007)

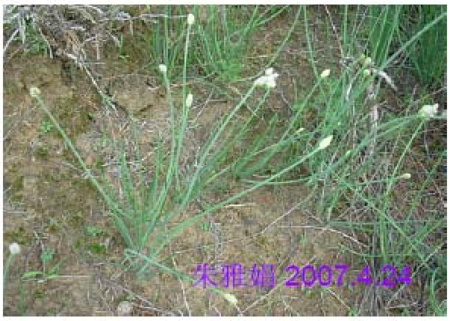

蒙古韭的全株

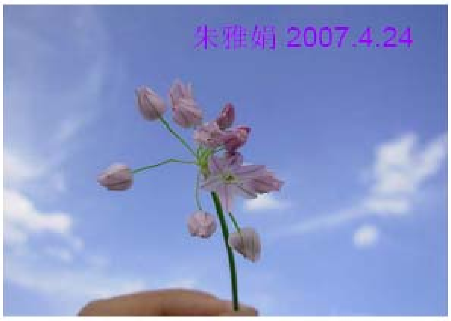

蒙古韭的花序

Fig. S17 Photographs of *Allium mongolicum* Regel. (Zhu Yajuan, 2007)

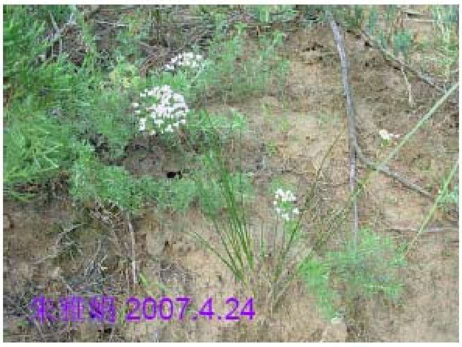

细叶韭的全株

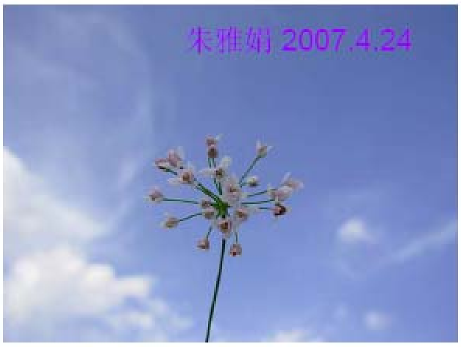

细叶韭的花序

Fig. S18 Photographs of *Allium tenuissimum* L. (Zhu Yajuan, 2007)

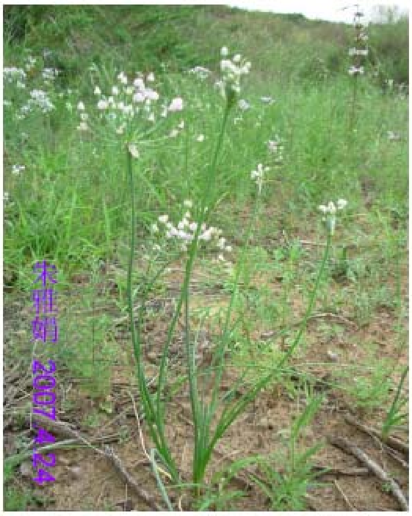

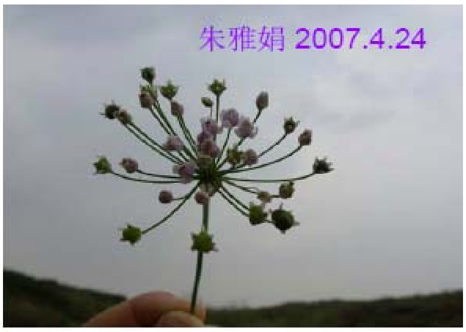

矮韭的花序

矮韭的全株

Fig. S19 Photographs of *Allium anisopodium* Ledeb. (Zhu Yajuan, 2007)

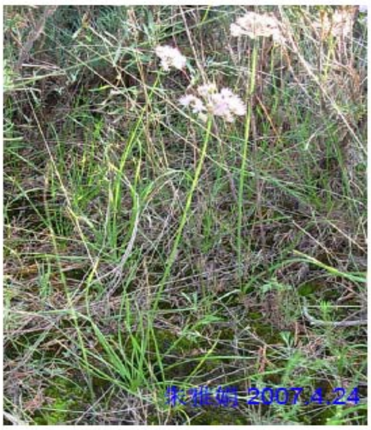

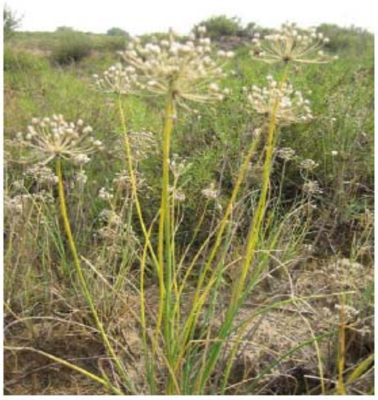

Fig. S20 Photographs of *Allium platystylum* Regel. (Zhu Yajuan, 2007)

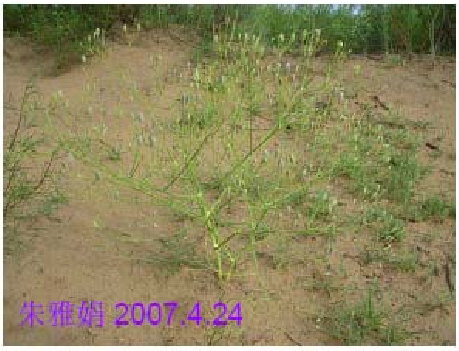

沙茴香的全株

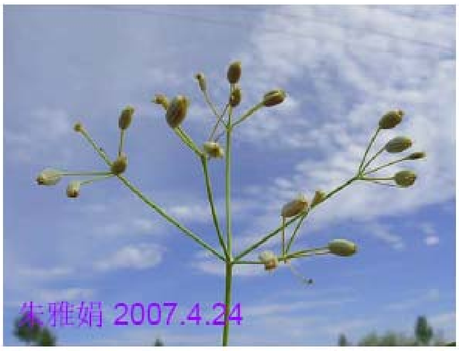

沙茴香的果序

Fig. S21 Photographs of *Ferula bungeana* Kitag. (Zhu Yajuan, 2007)

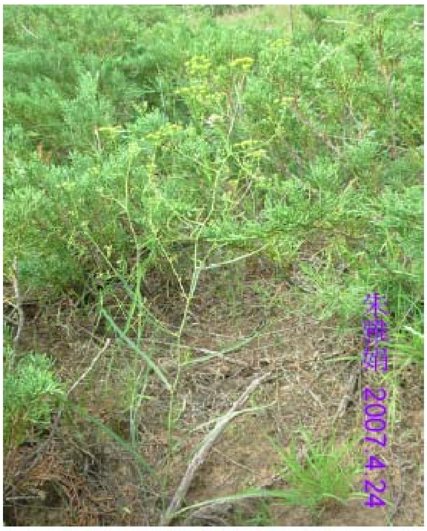

红柴胡的全株

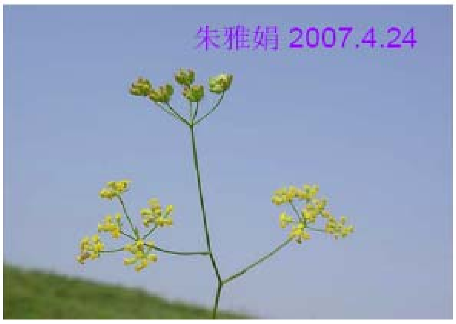

红柴胡的花序

Fig. S22 Photographs of *Bupleurum bicaule* Helm. (Zhu Yajuan, 2007)

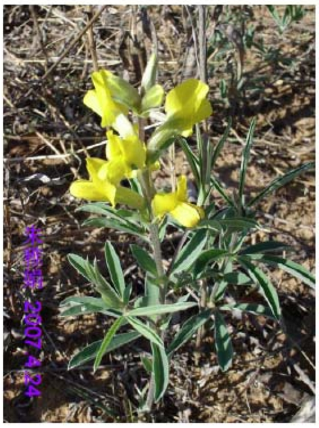

披针叶黄华的花枝

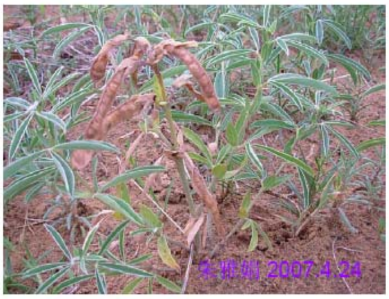

披针叶黄华的果枝

Fig. S23 Photographs of *Thermopsia laceolata* R. Br. (Zhu Yajuan, 2007)

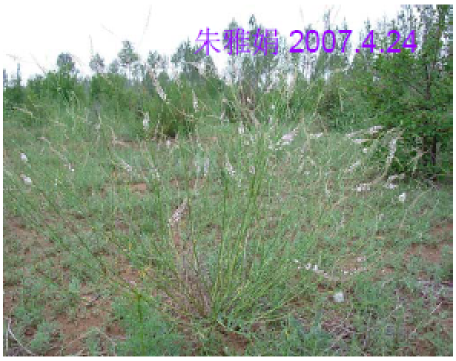

草木樨状黄芪的全株

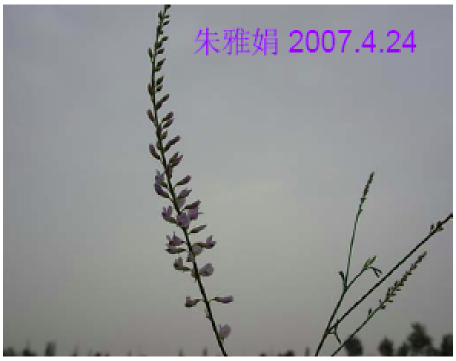

草木樨状黄芪的花序

Fig. S24 Photographs of *Astragalus melilotoides* Pall. (Zhu Yajuan, 2007)

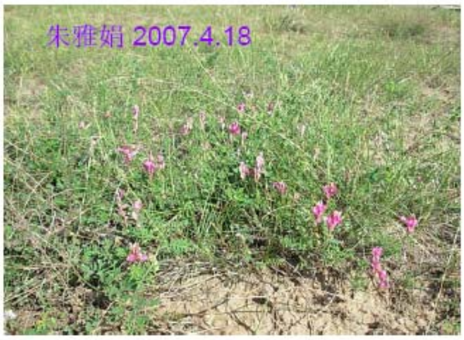

华北岩黄芪的全株

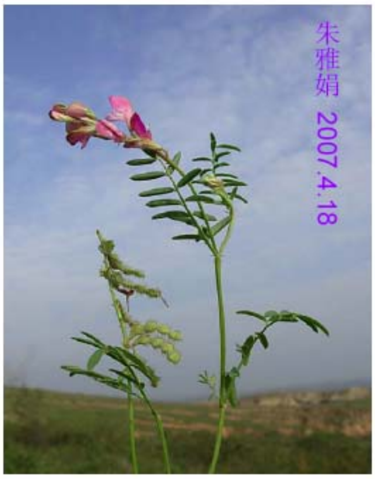

华北岩黄芪的花果

Fig. S25 Photographs of *Hedysarum gmelinii* Ledeb. (Zhu Yajuan, 2007)

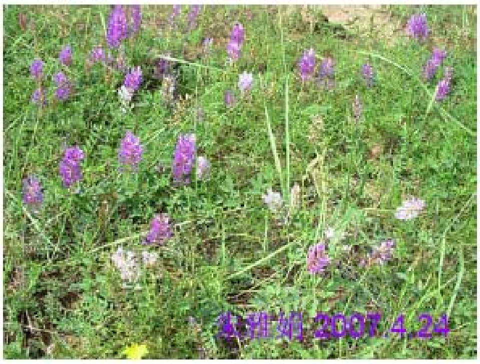

斜茎黄芪的全株

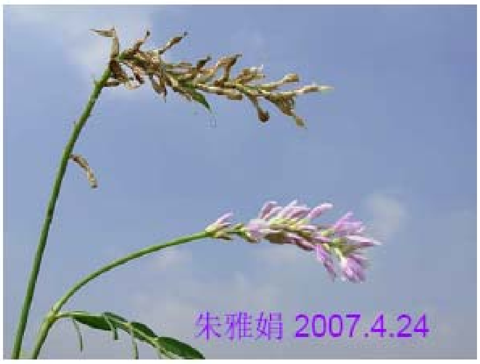

斜茎黄芪的花序

Fig. S26 Photographs of Astragalus adsurgens Pall. (Zhu Yajuan, 2007)

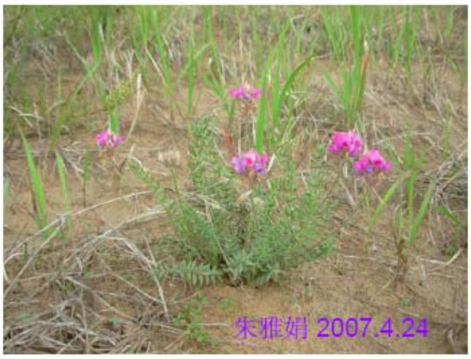

沙珍棘豆的全株

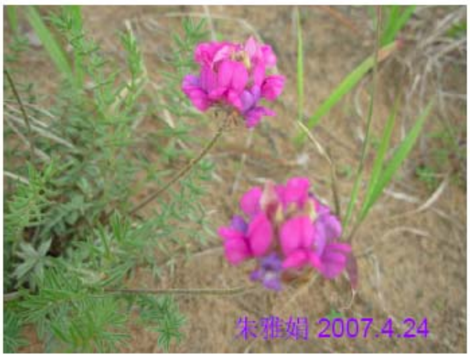

沙珍棘豆的花序

Fig. S27 Photographs of *Oxytropis psammocharis* Rance. (Zhu Yajuan, 2007)

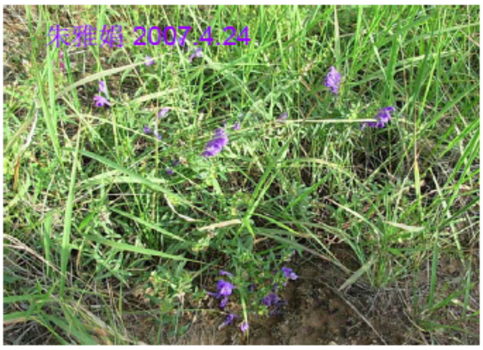

并头黄芩的全株

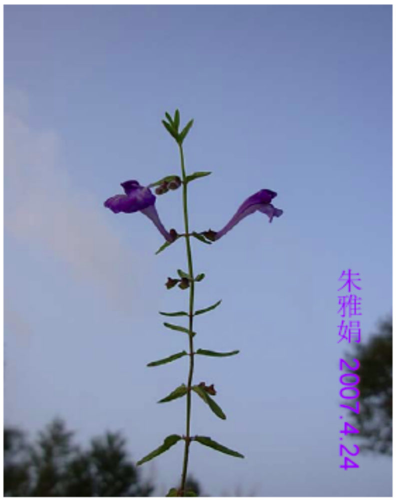

并头黄芩的花序

Fig. S28 Photographs of *Scutellaria scordifolia* Fisch. Ex Schrenk. (Zhu Yajuan, 2007)

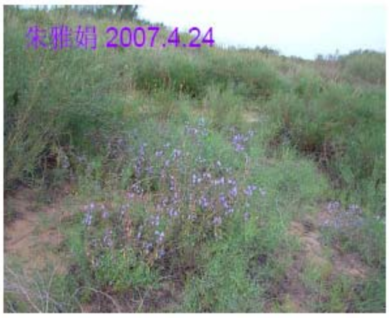

香青兰的全株

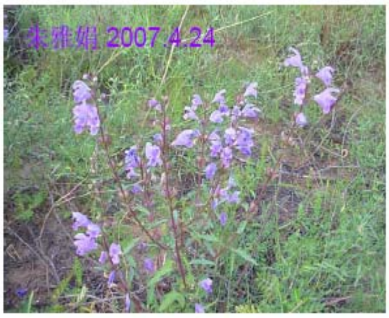

香青兰的花枝

Fig. S29 Photographs of Dracocephalum moldavica Linn. (Zhu Yajuan, 2007)

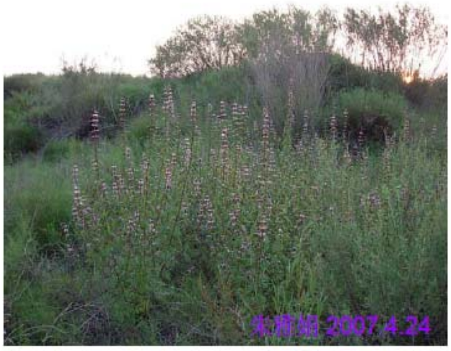

细叶益母草的全株

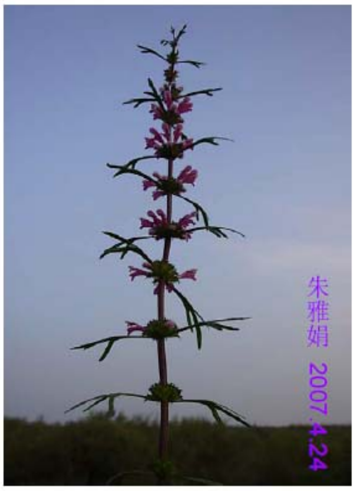

细叶益母草的花序

Fig. S30 Photographs of *Leonurus sibiricus* Linn. (Zhu Yajuan, 2007)

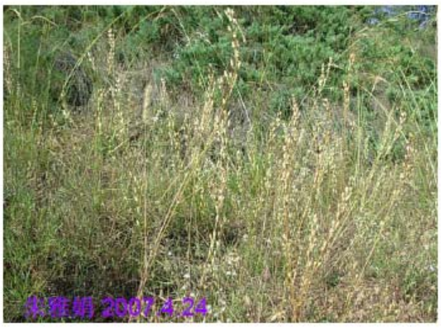

毛萼麦瓶草的全株

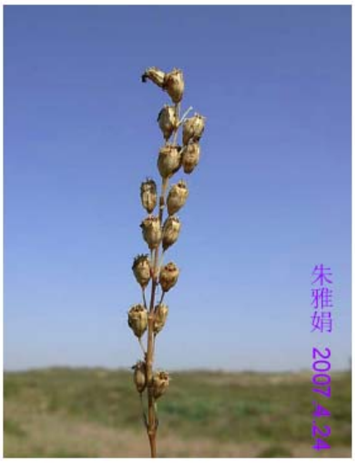

毛萼麦瓶草的果序

Fig. S31 Photographs of *Silene repens* Patr. (Zhu Yajuan, 2007)

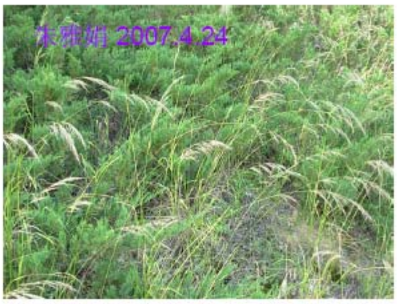

羽茅的全株

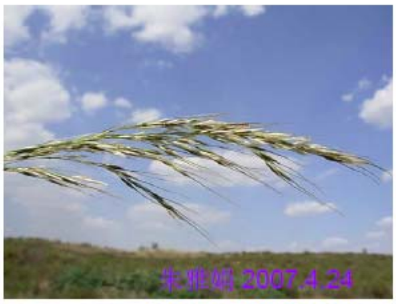

羽茅的花序

Fig. S32 Photographs of *Achnatherum sibiricum* (Linn.) Keng. (Zhu Yajuan, 2007)

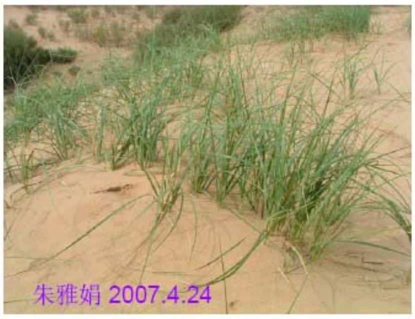

沙鞭的分株

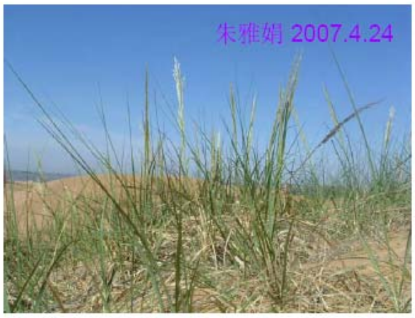

沙鞭的果序

Fig. S33 Photographs of *Psammochloa villosa* (Trin.) Bor. (Zhu Yajuan, 2007)

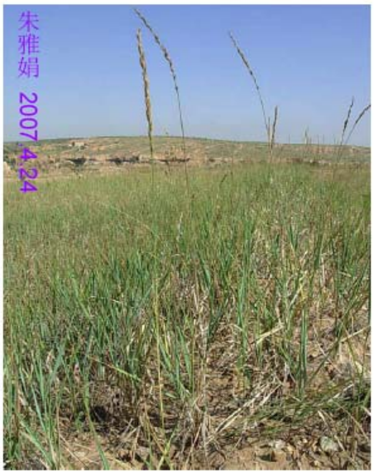

披碱草的全株

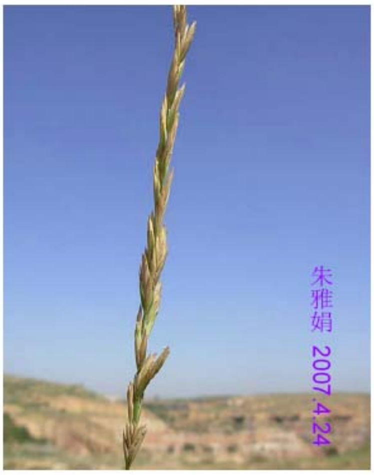

披碱草的果序

Fig. S34 Photographs of *Elymus dahurius* Turcz. (Zhu Yajuan, 2007)

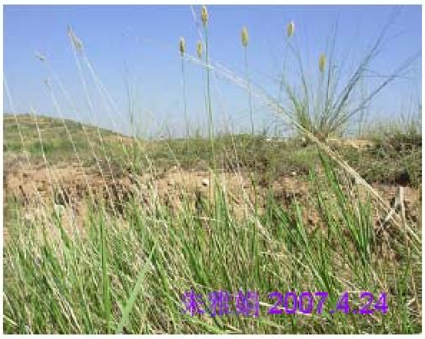

米氏冰草的全株

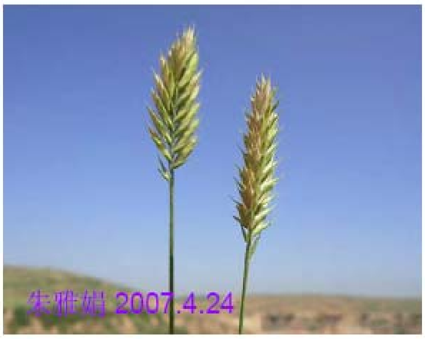

米氏冰草的花序

Fig. S35 Photographs of *Agropyron michnoi* Roshev. (Zhu Yajuan, 2007)

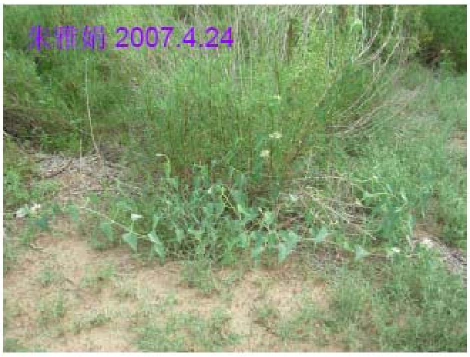

鹅绒藤的全株

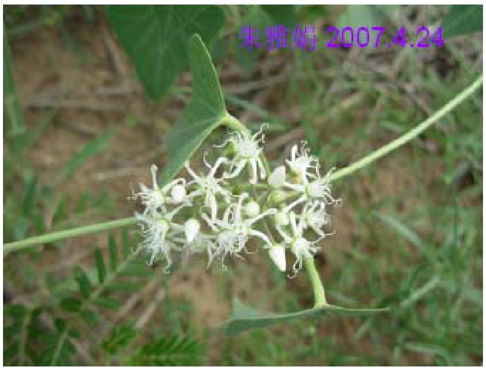

鹅绒藤的花序

Fig. S36 Photographs of *Cynanchum chinense* R. Br. (Zhu Yajuan, 2007)

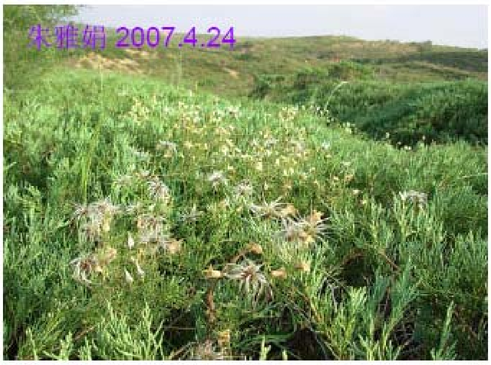

芹叶铁线莲的全株

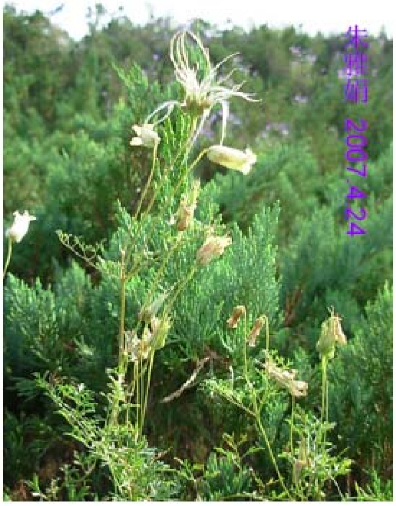

芹叶铁线莲的花果

Fig. S37 Photographs of Clematis aethusifolia Turcz. (Zhu Yajuan, 2007)

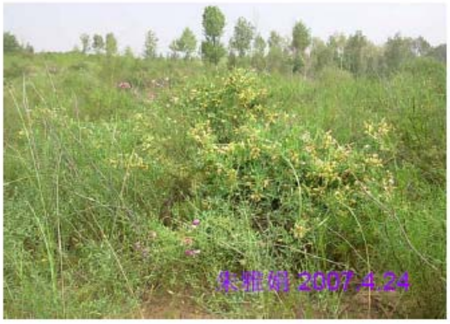

黄花铁线莲的全株

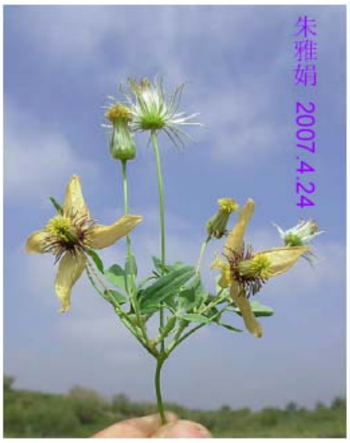

黄花铁线莲的花枝

Fig. S38 Photographs of *Clematis intricata* Bunge. (Zhu Yajuan, 2007)

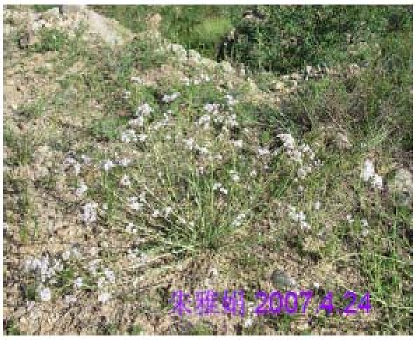

尖叶丝石竹的全株

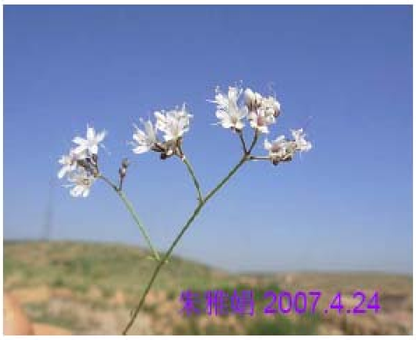

尖叶丝石竹的花序

Fig. S39 Photographs of *Gypsophila acutifolia* Fisch. (Zhu Yajuan, 2007)

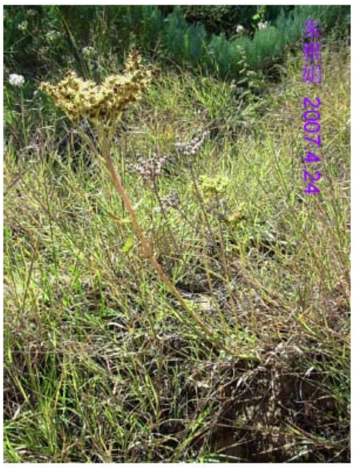

景天三七的全株

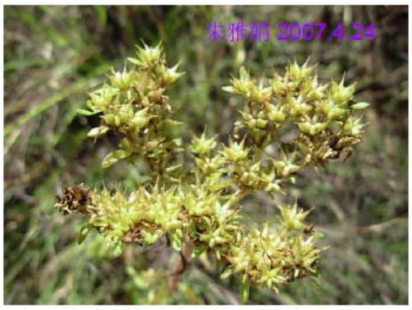

景天三七的果序

Fig. S40 Photographs of *Sedum aizoon* L. (Zhu Yajuan, 2007)

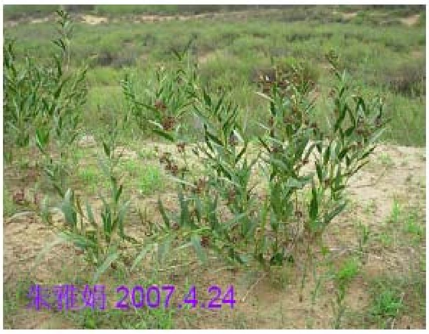

牛心朴子的全株

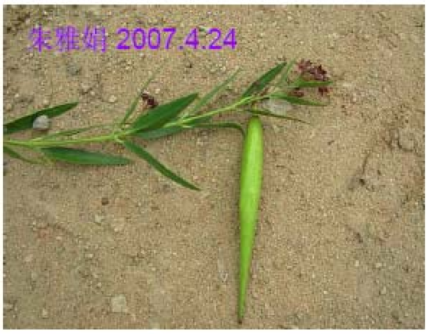

牛心朴子的花果

Fig. S41 Photographs of *Cynanchum Komarovii* Al lljinski (Zhu Yajuan, 2007)

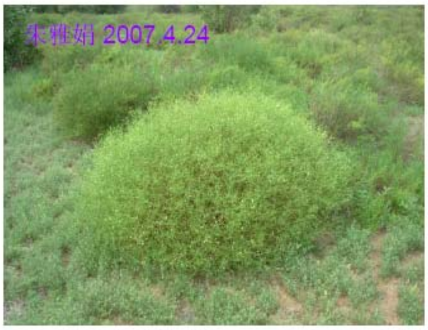

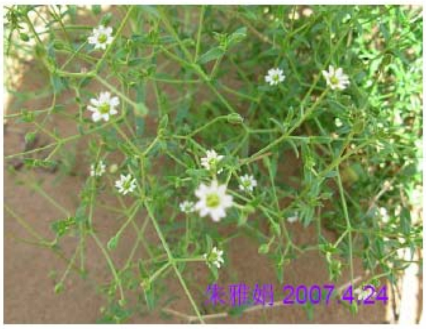

沙地繁缕的全株　　　　沙地繁缕的花枝

Fig. S42 Photographs of *Stellaria gypsophiloides* Fenl. (Zhu Yajuan, 2007)

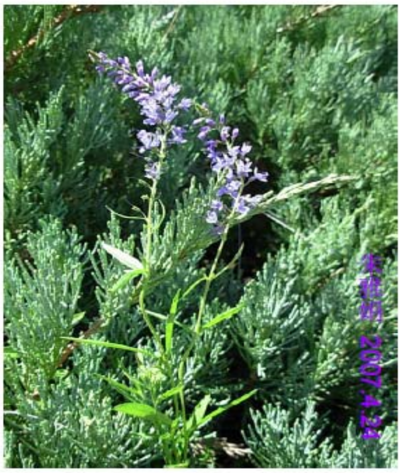

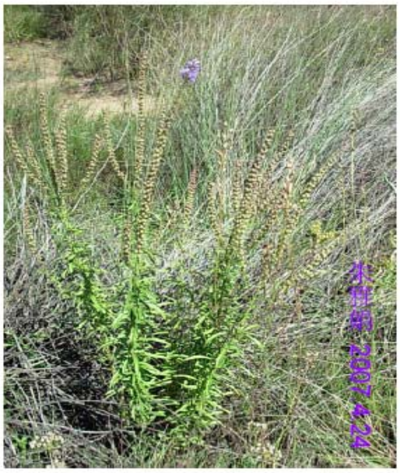

细叶婆婆纳的花序　　　　细叶婆婆纳的果序

Fig. S43 Photographs of *Veronica linarufolia* Pall. Ex Link. (Zhu Yajuan, 2007)

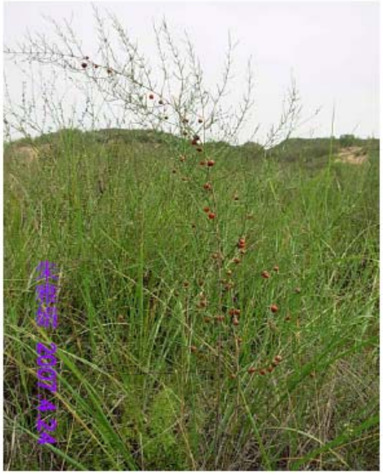

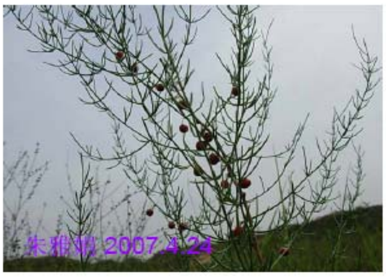

兴安天门冬的果枝

兴安天门冬的全株

Fig. S44 Photographs of *Asparagus dauricus* Fisch. Ex Link. (Zhu Yajuan, 2007)

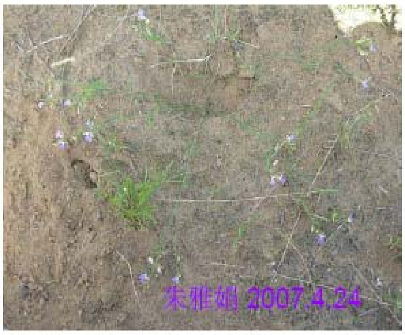

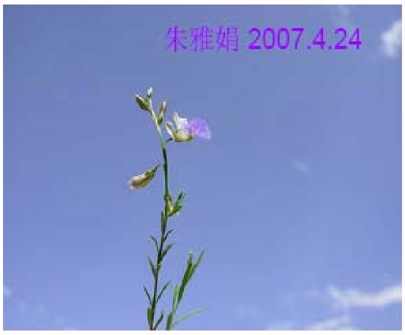

远志的全株　　　远志的花序

Fig. S45 Photographs of *Polygala tenuifolia* Willd. (Zhu Yajuan, 2007)

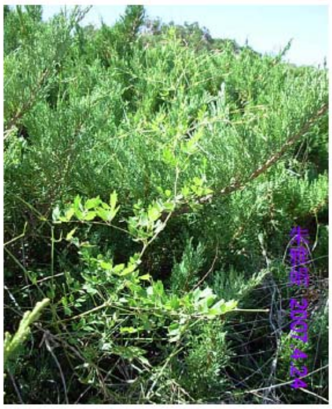

展枝唐松草的全株

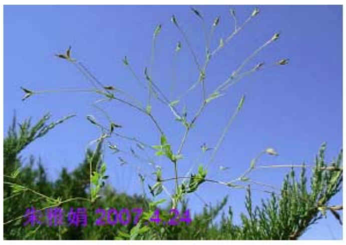

展枝唐松草的果序

Fig. S46 Photographs of *Thalictrum squarrosum* Steph. Ex Willd. (Zhu Yajuan, 2007)

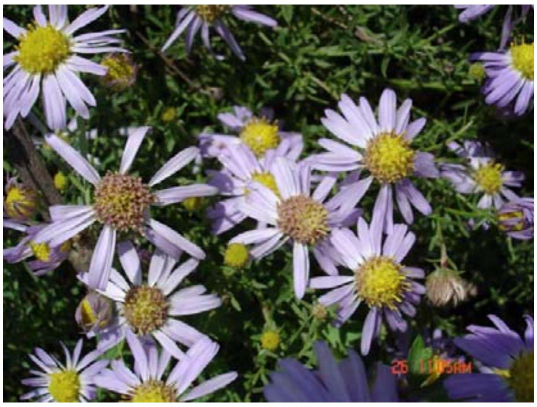

阿尔泰狗娃花

Fig. S47 Photographs of *Heteropappus altaicus* (Willd.) Novopokr (Zhu Yajuan, 2007)

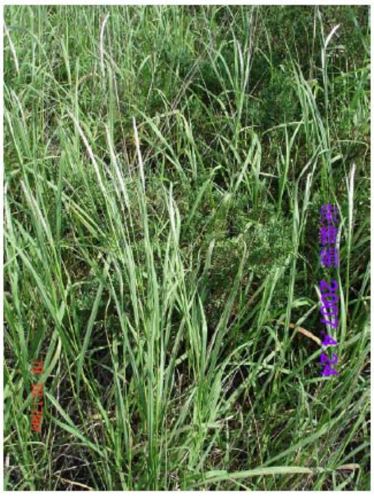

白草

Fig. S48 Photographs of *Pennisetum cenfrasiaticum* Tzvel. (Zhu Yajuan, 2007)

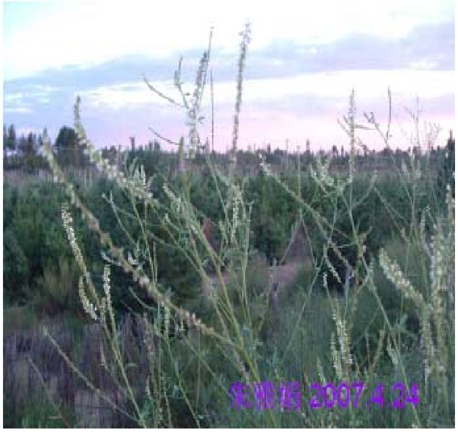

白花草木樨

Fig. S49 Photographs of *Melilotus alba Medic.* Ex Desr. (Zhu Yajuan, 2007)

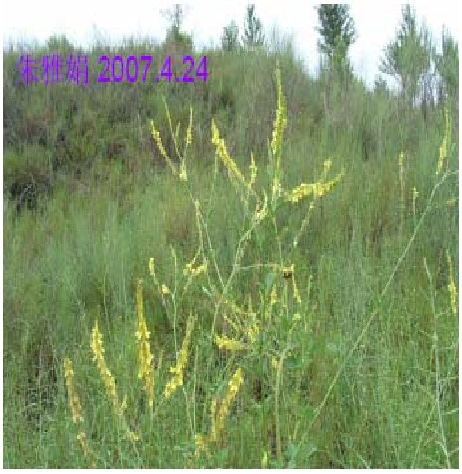

黄香草木樨

Fig. S50 Photographs of *Melilotus altissima* Thuill. (Zhu Yajuan, 2007)

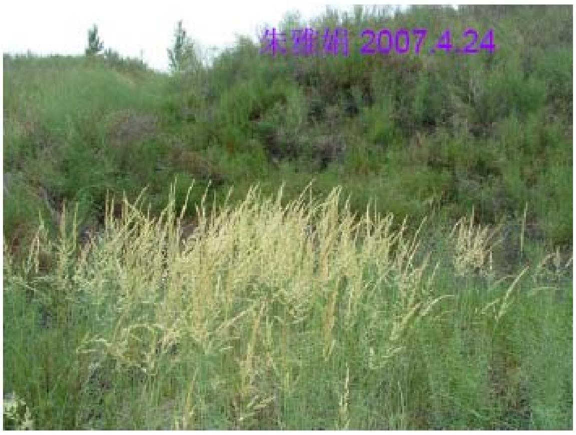

假苇拂子茅

Fig. S51 Photographs of *Calamagrostis pseudophragmites* (Hall. F.) Koel. (Zhu Yajuan, 2007)

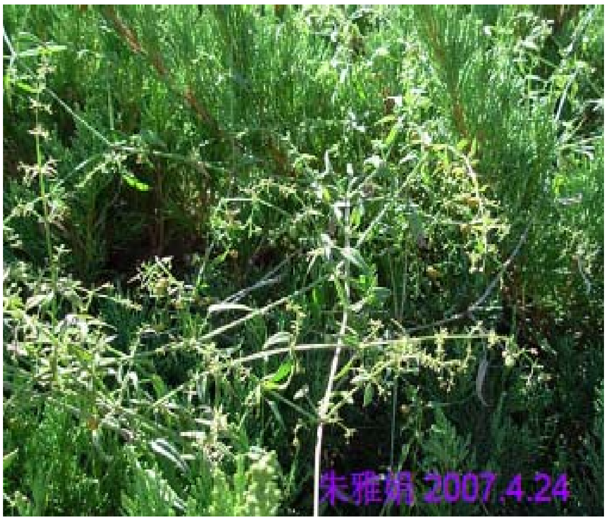

茜草

Fig. S52 Photographs of *Rubia manjith* Roxb. Ex Fleming. (Zhu Yajuan, 2007)

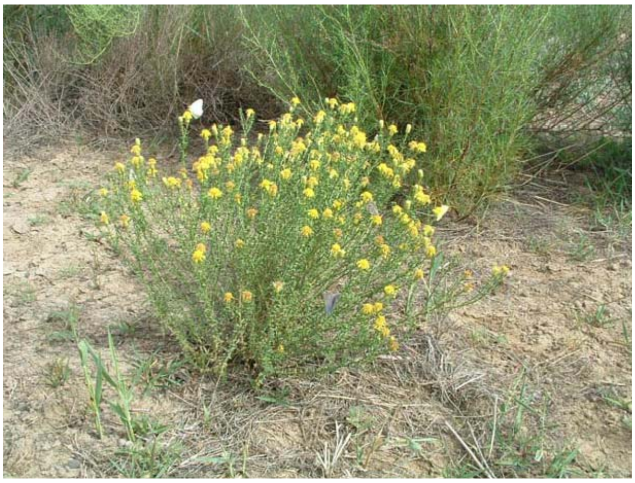

蓼子朴

Fig. S53 Photographs of *Inula salsolioides* (Turcz.) Ostenf. (Zhu Yajuan, 2007)

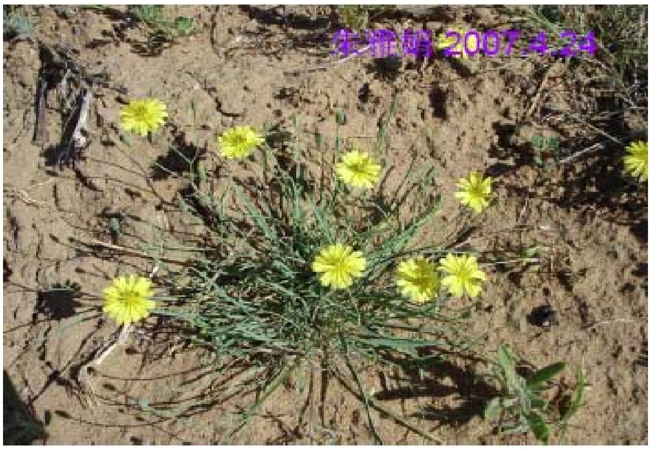

丝叶山苦荬

Fig. S54 Photographs of *Ixeris chinensis* (Thunb.)Nakai var. graminifolia (Ledeb.) H. C. Fu (Zhu Yajuan, 2007)

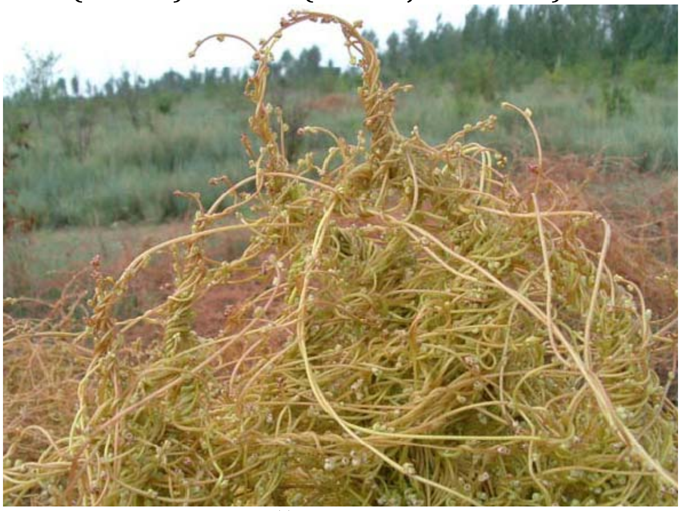

菟丝子

Fig. S55 Photographs of *Cuscuta chinensis* Lam. (Zhu Yajuan, 2007)

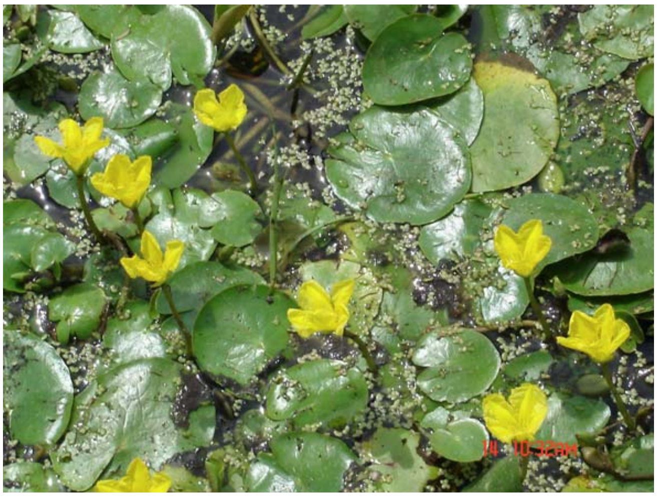

荇菜

Fig. S56 Photographs of *Nymphoides peltatum* (Gmel.) O. Kuntze. (Zhu Yajuan, 2007)

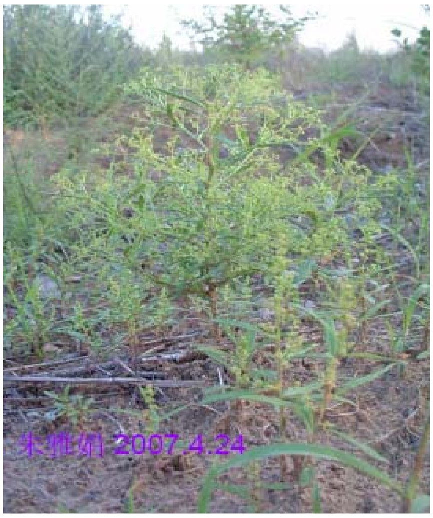

刺藜

Fig. S57 Photographs of *Chenopodium aristatum* L.(Zhu Yajuan, 2007)

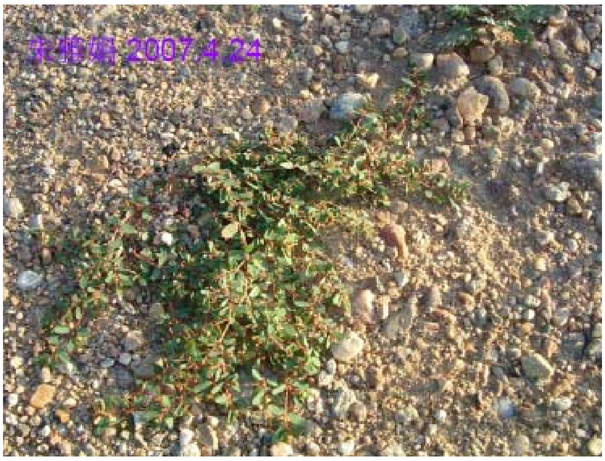

地锦

Fig. S58 Photographs of *Euphorbia humifusa* Willd. (Zhu Yajuan, 2007)

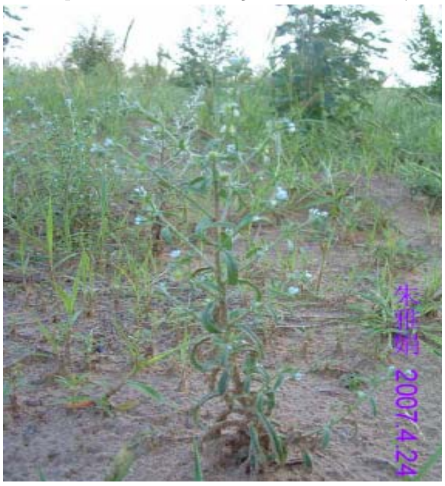

鹤虱

Fig. S59 Photographs of *Lappula myosopis* V. Wolf. (Zhu Yajuan, 2007)

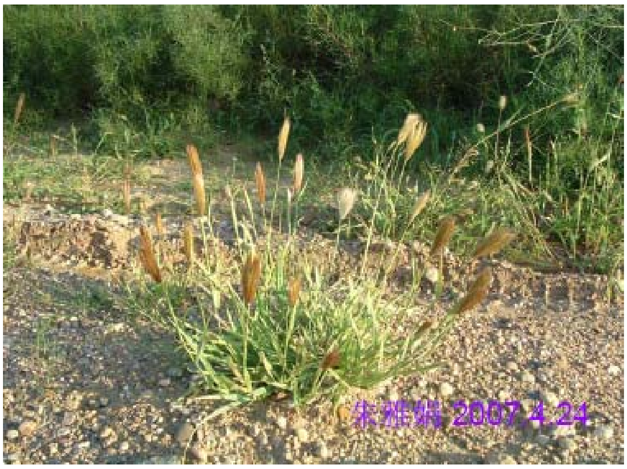

虎尾草

Fig. S60 Photographs of *Chloris virgata* Swartz. (Zhu Yajuan, 2007)

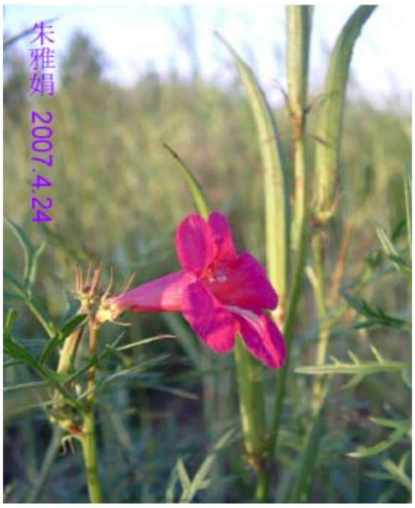

角蒿

Fig. S61 Photographs of *Incarvillea sinensis* Lam. (Zhu Yajuan, 2007)

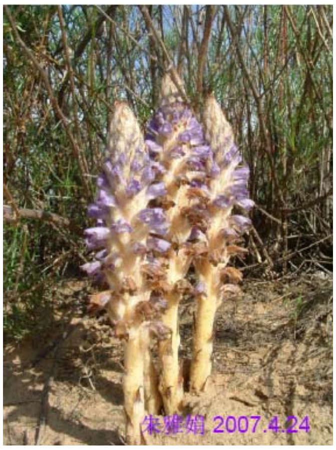

列当

Fig. S62 Photographs of *Orobanche caerulescens* Steph. (Zhu Yajuan, 2007)

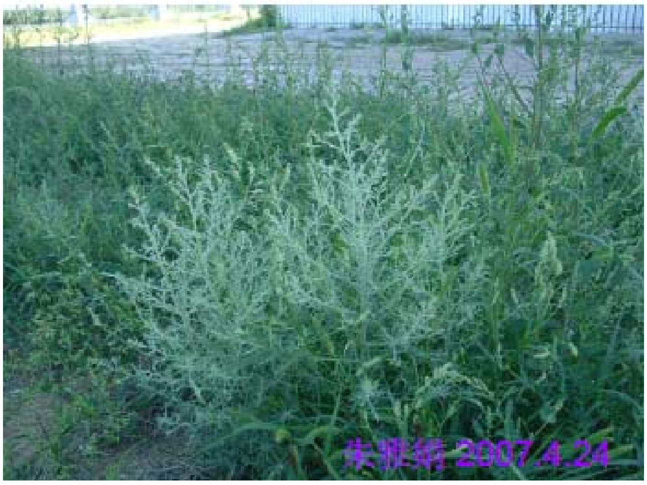

雾冰藜

Fig. S63 Photographs of *Bassia dasyphylla* (Fisch. Et Mey.) O. Kuntze. (Zhu Yajuan, 2007)

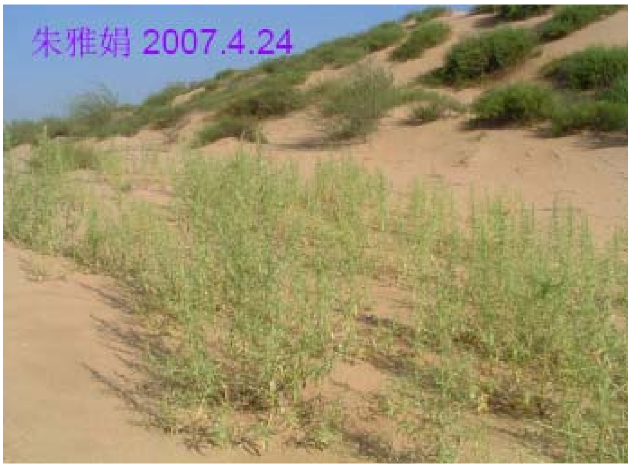

沙蓬

Fig. S64 Photographs of *Agriophyllum squarrosum* (L.) Moq. (Zhu Yajuan, 2007)

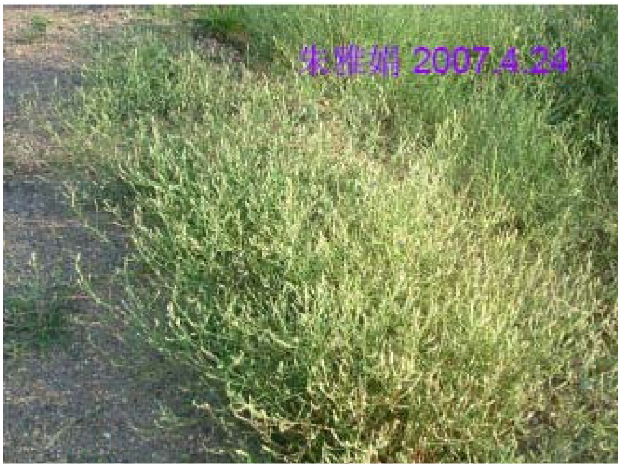

蒙古虫实

Fig. S65 Photographs of *Corispermum mongolicum* lljin. (Zhu Yajuan, 2007)

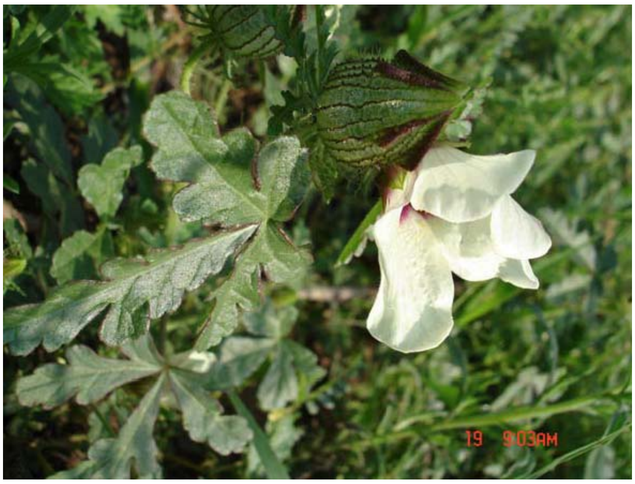

野西瓜苗

Fig. S66 Photographs of *Hibiscus trionum* Linn. (Zhu Yajuan, 2007)

Supplementary data

Shallow soil profile (1 m)								
Total nitrogen (mg kg-1)			Ammonium (mg kg-1)			Nitrate (mg kg-1)		
	Fixed dune	s.d. values	Depth	Fixed dune	s.d. values	Depth	Fixed dune	s.d. values
10	331.37	1.01	10	5.60	0.017	10	5.38	0.44
20	116.39	16.63	20	3.48	0.157	20	1.24	0.024
30	93.66	12.74	30	3.47	0.124	30	1.03	0.241
40	73.33	13.32	40	3.09	0.134	40	0.81	0.089
50	81.00	11.53	50	3.02	0.348	50	0.80	0.105
60	84.28	4.24	60	3.15	0.165	60	0.66	0.128
70	88.23	2.36	70	3.34	0.036	70	0.56	0.057
80	81.63	2.24	80	3.30	0.044	80	0.62	0.189
90	81.19	1.06	90	3.25	0.008	90	0.62	0.282
100	77.04	2.06	100	3.25	0.027	100	0.70	0.248
Depth	Semi-fixed dune	sd values	Depth	Semi-fixed dune	sd values	Depth	Semi-fixed dune	sd values
10	157.95	67.28	10	3.39	0.039	10	1.11	0.374
20	84.67	14.01	20	3.16	0.16	20	1.03	0.373
30	64.33	14.55	30	2.96	0.149	30	0.86	0.235
40	58.83	10.78	40	2.95	0.116	40	0.73	0.128
50	59.33	10.01	50	2.95	0.099	50	0.63	0.069
60	43.30	10.35	60	2.81	0.128	60	0.58	0.049
70	51.05	4.02	70	2.92	0.058	70	0.47	0.143
80	31.02	2.38	80	2.81	0.03	80	0.62	0.143
90	38.97	7.92	90	2.89	0.137	90	0.56	0.032
100	32.62	4.79	100	2.85	0.046	100	0.40	0.001
Depth	Mobile dune	s.d. values	Depth	Mobile dune	s.d. values	Depth	Mobile dune	s.d. values
10	52.66	2.52	10	3.00	0.528	10	0.76	0.28
20	51.67	17.95	20	2.98	0.038	20	0.53	0.17
30	49.67	9.82	30	2.97	0.036	30	0.49	0.21
40	45.33	13.80	40	2.92	0.02	40	0.48	0.14
50	43.46	23.31	50	2.90	0.035	50	0.42	0.10
60	38.33	18.61	60	2.86	0.035	60	0.40	0.08
70	38.00	9.64	70	2.85	0.041	70	0.58	0.22
80	35.00	6.93	80	2.82	0.014	80	0.52	0.17
90	34.33	8.14	90	2.82	0.063	90	0.52	0.33
100	34.77	3.91	100	2.83	0.022	100	0.49	0.01

Deep soil profile (5 m)					
soil nitrate (mg kg^{-1})					
Depth	Fixed dune	depth	Semi-fixed dune	Depth	Mobile dune
20	3.27	20	1.12	20	0.63
40	1.72	40	1.12	40	0.66
60	0.68	60	1.14	60	0.66
80	0.70	80	0.77	80	0.46
100	1.25	100	0.70	100	0.45
120	1.36	120	0.21	120	0.45
140	1.25	140	0.15	140	0.61
160	0.83	160	0.19	160	0.67
180	0.50	180	0.26	180	0.68
200	0.63	200	0.29	200	0.67
220	0.70	220	0.30	220	0.74
240	0.74	240	0.43	240	0.77
260	0.50	260	0.52	260	0.76
280	0.39	280	0.79	280	0.66
300	0.61	300	0.66	300	0.67
320	0.79	320	0.46	320	0.54
340	1.58	340	0.39	340	0.63
360	1.54	360	0.32	360	1.21
380	1.45	380	0.35	380	1.89
400	1.32	400	0.43	400	3.09
420	1.43	420	0.37	420	3.84
440	2.38	440	0.33	440	3.29
460	1.85	460	0.31	460	2.54
480	1.87	480	0.31	480	3.64
500	1.93	500	0.32	500	3.80
